# High-Performance Poly(vinylidene fluoride-hexafluoropropylene)-Based Composite Electrolytes with Excellent Interfacial Compatibility for Room-Temperature All-Solid-State Lithium Metal Batteries

**DOI:** 10.1021/acsomega.2c01338

**Published:** 2022-05-30

**Authors:** Si-Yuan Du, Guo-Xi Ren, Nian Zhang, Xiao-Song Liu

**Affiliations:** † State Key Laboratory of Functional Materials for Informatics, Shanghai Institute of Microsystem and Information Technology, Chinese Academy of Sciences, Shanghai 200050, China; ‡ Tianmu Lake Institute of Advanced Energy Storage Technologies, Liyang, Jiangsu 213300, China; § University of the Chinese Academy of Sciences, Beijing 100049, China; ∥ School of Physical Science and Technology, Shanghai Tech University, Shanghai 201210, China; ⊥ National Synchrotron Radiation Laboratory, University of Science and Technology of China, Hefei, Anhui 230029, China

## Abstract

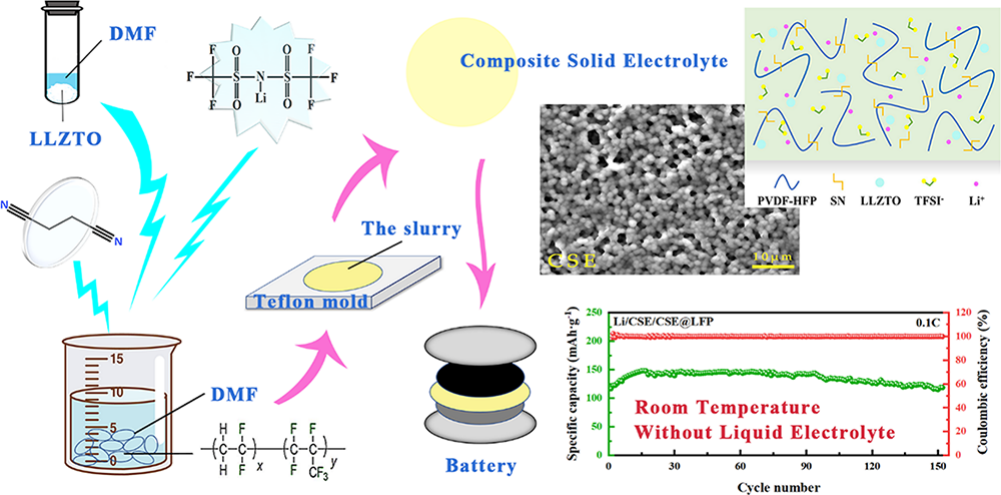

Composite solid-state electrolytes (CSEs) have been developed rapidly in recent years owing to their high electrochemical stability, low cost, and easy processing characteristics. Most CSEs, however, require high temperatures or flammable liquid solvents to exhibit their acceptable electrochemical performance. Room-temperature all-solid-state batteries without liquid electrolytes are still unsatisfactory and under development. Herein, we have prepared a composite solid electrolyte with excellent performance using a polymer electrolyte poly(vinylidene fluoride-hexafluoropropylene) and an inorganic electrolyte Li_6.4_La_3_Zr_1.4_Ta_0.6_O_12_. With the assistance of lithium salts and plasticizers, the prepared CSE achieves a high ionic conductivity of 4.05 × 10^–4^ S·cm^–1^ at room temperature. The Li/CSE/Li symmetric cell can be stably cycled for more than 1000 h at 0.1 mA/cm^2^ without short circuits. The all-solid-state lithium metal battery using a LiFePO_4_ cathode displays a high discharge capacity of 148.1 mAh·g^–1^ and a capacity retention of 90.21% after 100 cycles. Moreover, the high electrochemical window up to 4.7 V of the CSE makes it suitable for high-voltage service environments. The all-solid-state battery using a lithium nickel-manganate cathode shows a high discharge specific capacity of 197.85 mAh·g^–1^ with good cycle performance. This work might guide the improvement of future CSEs and the exploration of flexible all-solid-state lithium metal batteries.

## 1 Introduction

Lithium-ion batteries (LIBs) are considered to be one of the most promising batteries for next-generation electric vehicles and plug-in hybrid vehicles owing to the advantages of their high energy density, high efficiency, long cycle life, and environmental friendliness.^1−3^ These stringent and increasing needs put forward higher requirements for the energy density and safety performance of LIBs.^4−6^ However, traditional lithium-ion batteries often suffer from limited capacities, inadequate electrochemical stabilities, and serious safety problems owing to the use of volatile and flammable liquid electrolytes.^7,8^

Compared with traditional liquid batteries, all-solid-state batteries have attracted great attention due to their potential advantages of high weights and volume energy densities, wide operating temperature ranges, long cycle life, and excellent safety.^9−11^ The current mainstream solid electrolytes can be mainly classified into polymer solid electrolytes,^12−14^ sulfide solid electrolytes,^15,16^ and oxide solid electrolytes.^17,18^ Among them, solid garnet oxide electrolyte Li_7_La_3_Zr_2_O_12_ (LLZO) gains increasing attention because of its high ionic conductivity at room temperature (∼10^–3^ S cm^–1^) and wide electrochemical stability window (>5 V).^19−21^ However, the rigidity nature of LLZO limits its processing characteristics and causes poor interfacial contact between solid-state electrolytes and electrodes.^22−24^ Moreover, lithium dendrites can still dilapidate oxide solid electrolytes due to the inhomogeneous dissolution and deposition of lithium, causing a severe short circuit of the battery.^25−27^

The excellent stability and flexibility of polymer solid electrolytes can play a good complementary role to LLZO,^28−31^ which stimulates intensive interest in the corresponding composite solid-state electrolytes (CSEs). Among polymer solid electrolytes, poly(vinylidene fluoride-hexafluoropropylene) (PVDF-HFP) is chosen owing to its slightly higher ionic conductivity, good thermal stability, and amorphous phase (-HFP).^32−35^ In CSEs, for one thing, PVDF-HFP can act as an excellent mechanical support for LLZO nanoparticles with excellent flexibility, which can significantly improve electrolyte/electrode interfacial contact and help inhibit the penetration of lithium dendrites.^36−38^ On the other hand, the introduction of LLZO can build new lithium-ion transmission pathways, which can greatly improve the lithium-ion conduction of polymer electrolytes.^39−42^ LLZO particles and the anion can also reduce the double-layer electric field between the lithium metal and the electrolyte to inhibit the electrochemical decomposition of PVDF-HFP.^43−45^

The CSEs built up by PVDF-HFP and LLZO are expected to combine their advantages and overcome the disadvantages of each other, facilitating the promising performance of the all-solid-state lithium batteries (ASSLBs) at room temperature. In this work, we used PVDF-HFP as the matrix and combined it with a small amount of Ta-doped LLZO (LLZTO, Li_6.4_La_3_Zr_1.4_Ta_0.6_O_12_) nanoparticles. We also added a certain amount of succinonitrile (SN) and lithium bis(trifluoromethanesulfonyl)imide (LiTFSI) to improve the performance of CSEs. The CSEs show high lithium-ion conductivity, low interfacial resistance, and good stability toward lithium metal. In addition, we applied the CSEs in Li|LFP (lithium iron phosphate) and Li|LNMO (lithium nickel manganese oxide) cells, which delivered excellent electrochemical properties and cycle performance without liquid electrolytes at room temperature. These results indicate the extraordinary electrochemical capabilities of this CSE and its promising applications in high-energy all-solid-state lithium metal batteries.

## 2 Materials and Methods

### 2.1 Preparation of the Composite Solid-State Electrolytes

**Figure 1:**
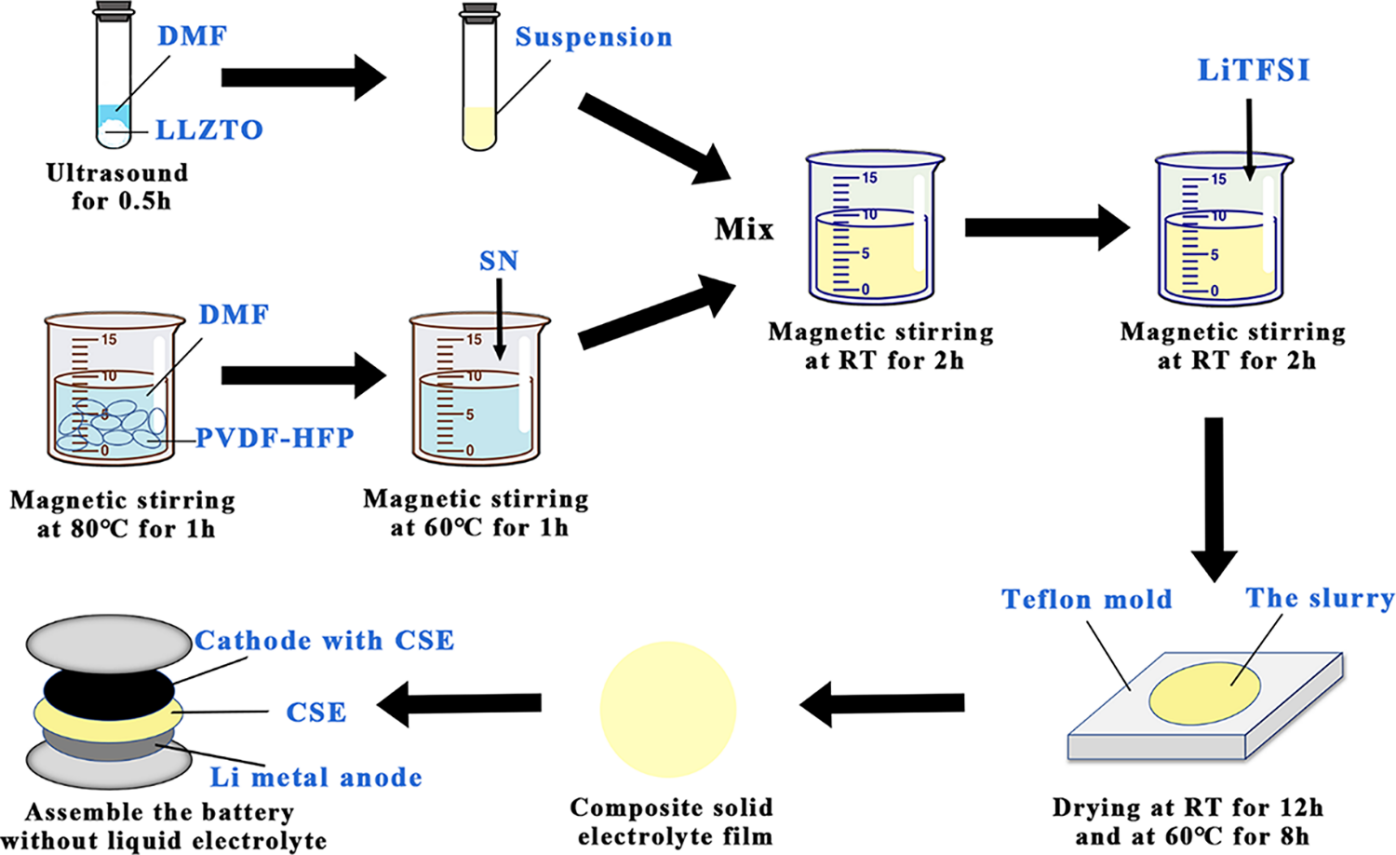
Schematic diagram of the preparation process of the composite solid-state electrolyte.

In order to obtain the optimal ratio of the composite solid-state electrolyte, we systematically studied the effects of different additive contents on the ionic conductivity. PVDF-HFP (Sigma-Aldrich, Mw ∼ 400,000), as a frame structure, was first dissolved in dimethylformamide (DMF) solution at 0.2 g/mL by magnetically stirring for 1 h at 80 °C. Subsequently, only LiTFSI with different mass fractions from 20 to 80% was added. The mixtures were magnetically stirred for 2 h at room temperature. Then, the slurry was casted on the Teflon mold (19 mm diameter round) and kept at room temperature for 12 h. To obtain a dense membrane, the obtained solid membrane was dried at 60 °C for 8 h. For the next step, the ratio of PVDF-HFP:LiTFSI was fixed at 6:4, which exhibits the highest ionic conductivity, and then SN with different mass fractions from 5 to 30% was added in the slurry. The mixed slurry was magnetically stirred at 60 °C for 1 h, and then the membrane preparation processes were repeated. Finally, on the basis of the optimal ratio for PVDF-HFP:LiTFSI:SN fixed at 6:4:2.5, LLZTO was dissolved in a small amount of DMF by ultrasonic dispersion and then added into the slurry. The mass fraction range for LLZTO was 6–18%. The mixtures were magnetically stirred for 2 h and were fabricated into membranes using the same method. After obtaining the optimal mass ratio of the four substances, the preparation processes of CSEs were further optimized and are shown in Figure 1. The thickness of the prepared CSE is about 300 μm. All the preparation steps are done in a glove box filled with argon.

### 2.2 Preparation of Composite Cathode Materials

To obtain the composite LFP cathode (CSE@LFP), LFP (Aladdin, Shanghai, China) and acetylene black (C) were uniformly dispersed in DMF solvent and magnetically stirred for 4 h. Then, the composite solid electrolyte slurry was added with the mass ratio LFP:C:CSE = 6:1:3. The mixture was magnetically stirred for another 2 h and casted onto Al foil, which was subsequently dried at 80 °C for 20 h. The CSE@LFP cathode was finally made into a 12 mm-diameter disc with an areal mass loading of about 2.76 mg/cm^2^. The composite LNMO cathode (CSE@LNMO) was acquired by the same method with an areal mass loading of about 3.15 mg/cm^2^. LNMO materials were obtained from Peking University Shenzhen Graduate School (Shenzhen, China).

### 2.3 Material Characterization

The crystal structures of the electrolyte membranes were analyzed by X-ray diffraction (XRD) using a DX-2700A diffractometer (Cu Kα = 1.5406 Å). The surface images and elemental mappings of the samples were acquired by scanning electron microscopy (SEM) on a JEOL JSM-7800F instrument. The accelerating voltage of energy dispersive spectroscopy (EDS) for elemental mappings was set to 15 kV. The Fourier transform infrared spectroscopy (FTIR) curves of the samples were obtained with a VERTEX-80V infrared spectrometer.

### 2.4 Cell Assembly and Electrochemical Measurements

The electrochemical measurements were conducted by 2025-type coin cell with a lithium metal electrode or a stainless steel (SS) blocking electrode. The SS/CSE/SS symmetrical cells were assembled to measure the ionic conductivity, and the Li/CSE/Li symmetrical cells were used to evaluate the stability of the solid electrolyte toward Li metal. Electrochemical impedance spectroscopy (EIS), current polarization, electrochemical cyclic voltammetry (CV), electrochemical linear polarization (LSV), and other tests were all performed on an EC-LAB-SP-200 electrochemical workstation. The cycle performance of the symmetrical cells and all-solid-state batteries was tested on a Neware (CT-4008) workstation. The all-solid-state batteries were assembled using CSE@LFP or CSE@LNMO as a cathode and Li foil as an anode.

## 3 Results and Discussion

**Figure 2:**
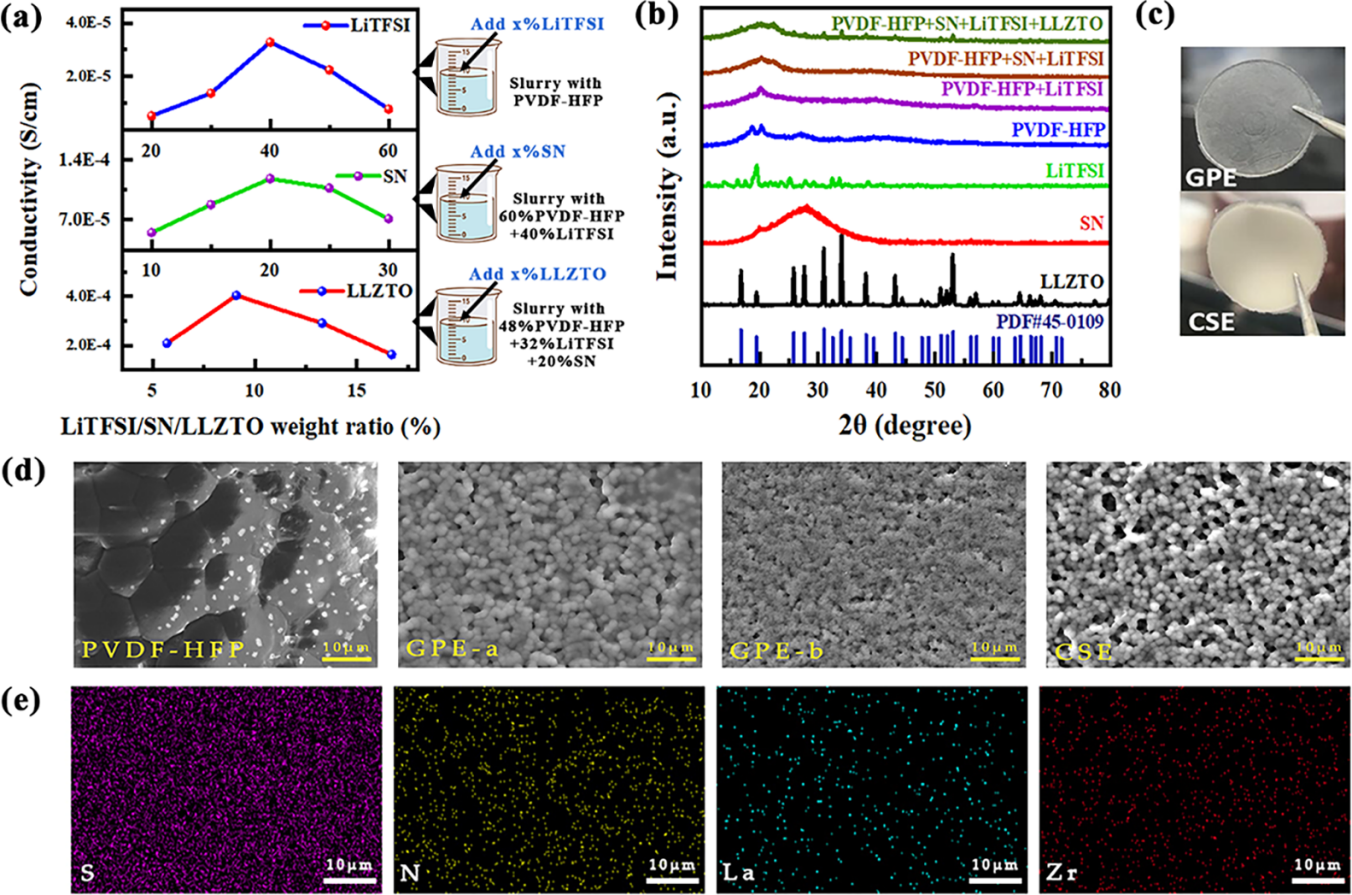
(a) Ionic conductivity at different contents of LiTFSI, SN, and LLZTO. (b) XRD patterns of the different membranes. PVDF-HFP, LiTFSI, SN, and LLZTO are presented as reference samples (PDF#45-0109 is the standard serial of cubic-phase LLZO). (c) Photographs of GPEs and CSEs. (d) SEM images of PVDF-HFP, GPE-a, GPE-b, and CSE (from left to right). (e) EDS elemental mappings of S, N, La, and Zr in CSE with an accelerating voltage of 15 kV.

The ionic conductivity of solid polymer electrolytes is generally 10^–9^–10^–6^ S·cm^–1^ at room temperature.^46^ To increase the ionic conductivity of CSEs, the effects of different additive ratios were systematically investigated. Figure 2a shows the variations of the ionic conductivity with different LiTFSI, SN, and LLZTO contents. We first synthesized PVDF-HFP-based solid gel polymer electrolytes (GPE) containing 20–80 wt % LiTFSI without SN and LLZTO. The concentration of lithium salt mainly affects the distribution of charge carriers and the long-range migration ability of lithium ions in the polymer. When the concentration of lithium salt is maintained at the low standard, it can be fully solubilized by PVDF-HFP, providing free lithium ions as charge carriers, which increases with lithium salt content.^47,48^ However, with the increase in lithium content to a certain extent, the solubilization effect is limited, and the electrostatic interactions between Li^+^ and TFSI^–^ become significant, which reduce the number of effective carriers and the ionic conductivity.^49^ Moreover, lithium salt compresses the space for free chain segment movement of the polymer and reduces the film-forming ability to the extent that the slurry cannot form a film when the content of lithium salt is too high. Thus, when the optimal weight ratio was adjusted to PVDF-HFP:LiTFSI = 6:4 (labeled as GPE-a), the highest ionic conductivity of 3.28 × 10^–5^ S·cm^–1^ was reached.

On that basis, we fixed the weight ratio of PVDF-HFP:LiTFSI = 6:4 and further added plasticizers to reduce the crystallinity of the polymer, which could enhance the chain segment motion of the polymer to facilitate the migration of lithium ions.^48,50^ SN is chosen as the plasticizer owing to its ability to weaken the van der Waals forces of the PVDF-HFP macromolecules and the hydrogen bonds between the chain segments. As shown in the X-ray diffraction patterns in Figure 2b, the peak intensity of PVDF-HFP is significantly weakened after the introduction of SN. The morphology in Figure 2d confirms that the plasticizer causes the disappearance of the large-sized PVDF-HFP crystals. SN also possesses a strong polar −C≡N groups, which can promote the dissolution and dissociation of lithium salts through intermolecular interactions.^51,52^ However, an excessive percentage of plasticizer not only weakens the polymer viscosity but may also drive toward partial loss of plastic-crystal order locally and more complex phenomena.^53^ The highest ionic conductivity of 1.18 × 10^–4^ S·cm^–1^ was achieved when SN was added around 20 wt %, and the corresponding PVDF-HFP:LiTFSI:SN is 6:4:2.5 (labeled as GPE-b).

Finally, we fixed PVDF-HFP:LiTFSI:SN = 6:4:2.5 and added LLZTO to further enhance the ionic conductivity. When 9.1 wt % LLZTO is added, the highest ionic conductivity of 4.05 × 10^–4^ S·cm^–1^ is achieved (labeled as CSE). The resulting composite electrolyte membrane is flexible and can be cut into desired shapes, as shown in Figure 2c. The SEM images and EDS elemental mappings of the membrane are shown in Figure 2d,e; microsized LLZTO particles are uniformly distributed in the CSE, providing continuous jump sites for Li^+^. The interfaces between PVDF-HFP and LLZTO also create high-speed lithium ion transmission channels.^54^ Thus, the ability of long-range migration of lithium ions is significantly improved. However, the uniformly distributed LLZTO powders are agglomerated with the further increase in inorganic fillers, leading to the disconnection of consecutive lithium-ion channels.^55,56^ Therefore, the optimal composition of CSE was determined as 43.6 wt % PVDF-HFP, 29.1 wt % LiTFSI, 18.2 wt % SN, and 9.1 wt % LLZTO.

**Figure 3:**
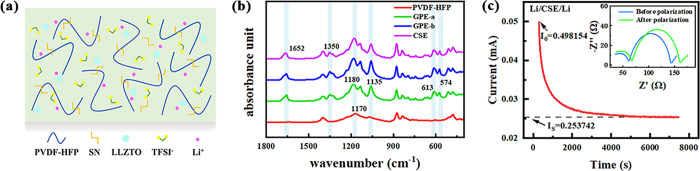
(a) Schematic diagram of CSE composite modification. (b) FTIR spectra of PVDF-HFP, GPE-a, GPE-b, and CSE. (c) Polarization curves of CSE at 10 mV DC voltage.

As shown in Figure 3a, the improvement of ionic conductivity may be attributed to the synergistic interaction of uniformly distributed amorphous PVDF-HFP, lithium salt, SN, and macrosized LLZTO. To investigate the partial interactions of different groups in the membrane, FTIR analysis was performed. Figure 3b shows the FTIR spectra of PVDF-HFP, which displays significant peaks at 1400, 1170, 1072, 873, 835, 511, and 480 cm^–1^. The peaks at 873 and 835 cm^–1^ can be assigned as the amorphous phase (β-phase) of PVDF-HFP, and the others are related to the crystalline phase (α-phase).^57^ After the introduction of LiTFSI, a new peak that appears at 1652 cm^–1^ can be assigned to the interaction of the C–H group and C–F group. The peaks at 574 and 1350 cm^–1^ are related to the N-CO-O symmetric stretching and asymmetric SO_2_ stretching modes, respectively. The peaks at 613 and 1135 cm^–1^ illustrate the interaction of Li^+^ with CF_2_ and CF_3_. There is a red-shift of the C–F group from 1170 to 1180 cm^–1^ mainly because the free Li^+^ inhibits the formation of hydrogen bonds between the groups.^58,59^ The newly formed interaction between functional groups suggests that new transport behaviors of lithium ions appear, while the similarity of the polymer’s spectra indicates that the original skeleton of the polymer matrix is still retained.^60^

Subsequently, we measured the direct current (DC) polarization curve and alternating current (AC) impedance of the Li/CSE/Li symmetrical cell at room temperature to demonstrate the effect of synergistic components to increase ionic mobility. As shown in Figure 3c, the CSEs provide a high lithium-ion mobility number of *t*_Li^+^_ = 0.43, which can be calculated from the following equation:
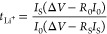
where Δ*V* is the applied polarization voltage of 10 mV, *I*_0_ and *R*_0_ are the initial current and electrolyte internal resistance before polarization, and *I*_S_ and *R*_S_ are the stable current and electrolyte internal resistance after polarization.

**Figure 4:**
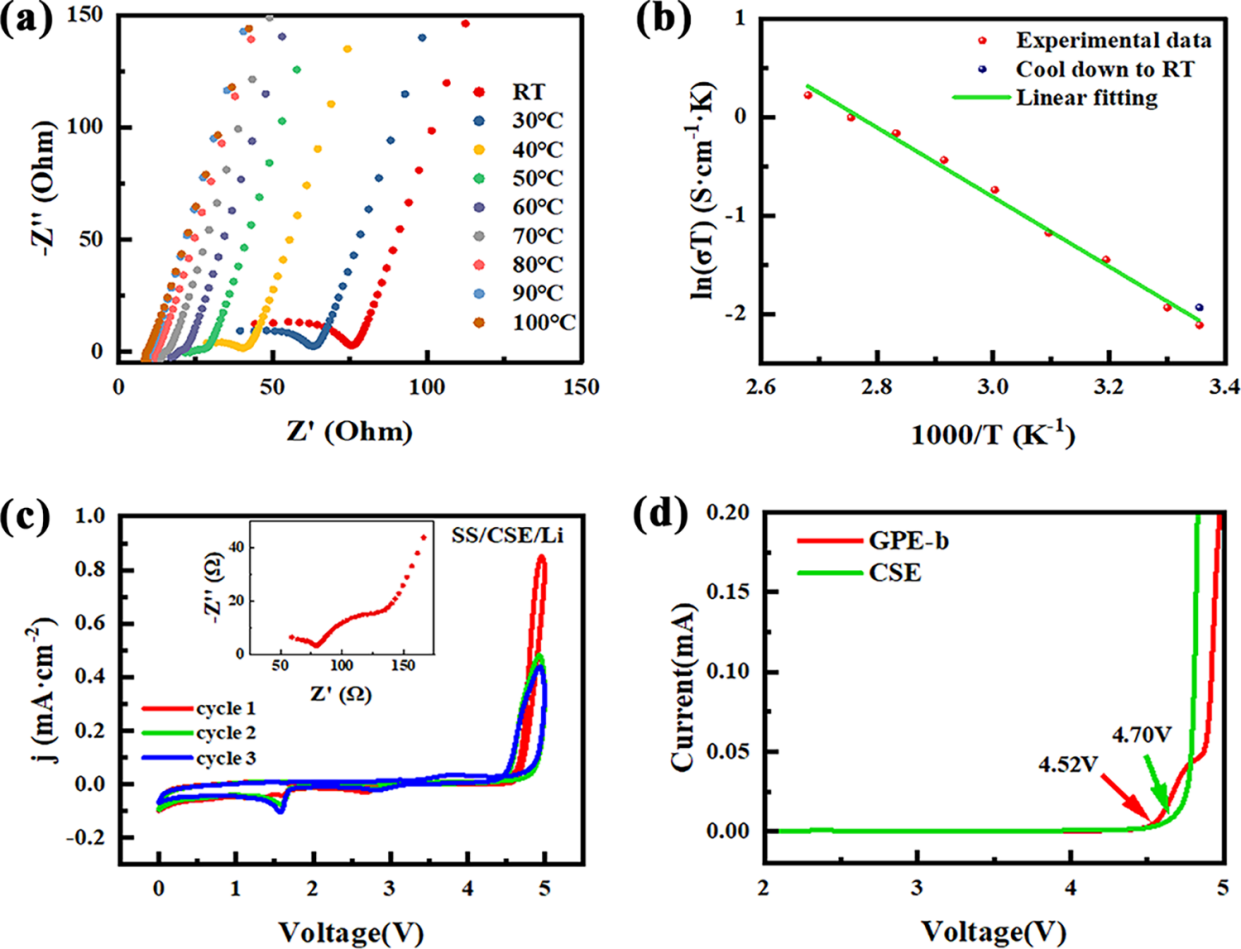
(a) EIS curves of CSE at different temperatures. (b) Arrhenius plots of CSE at temperatures ranging from 25 to 100 °C. (c) CV curves of CSE at the scan rate ν = 0.5 mV·s^–1^. (d) LSV curves of GPE-b and CSE.

The EIS of SS/CSE/SS symmetrical cell at different temperatures are shown in Figure 4a. After fitting according to the equivalent circuit shown in Figure S1,^61^ the calculated ionic conductivity is displayed in Figure S2. With the increasing of the temperature, the ionic conductivity of CSE enhances, which reaches 1.34 × 10^–3^ S·cm^–1^ at 60 °C and up to 3.16 × 10^–3^ S·cm^–1^ at 100 °C. The linear fitting result between temperature and conductivity exhibits a typical Arrhenius-type behavior shown in Figure 4b. The calculated activation energy (*E*_a_) is 0.207 eV, which is lower than that of pure PVDF-HFP, indicating the higher migration efficiency of Li^+^ and the stronger interaction between various additives and PVDF-HFP. The results are in agreement with the previous characterizations that the CSEs have low crystallinity, high ionic conductivity, and uniform phase distribution, which are beneficial for LIB applications.

In addition to ionic transport, the electrochemical stability window is also one of the important parameters to evaluate solid-state electrolytes, which can be determined using a Li/electrolyte/SS cell. The electrochemical window of polymer electrolytes is generally not adequate for commercial cathode materials, but the introduction of additives is valid to improve this situation.^48^ The CV curves of GPE-a containing only lithium salts and polymers are shown in Figure S3, which exhibits poor electrochemical stability and strong redox side reactions under applied voltage. In addition, the response current from GPE-a was the smallest among all samples, which is related to its extremely poor conductivity. The inherently huge impedance and the polarization impedance during the test combine to result in a very small response current. Also, the trend of the test current shows an obvious increase for most of the time, and thus it is difficult for us to identify the position of a specific redox peak. In contrast, smooth platforms and exact peak positions can be observed in CV records of GPE-b and CSE. As shown in Figure S4, the addition of SN enhances intermolecular interactions and greatly reduces the side reactions. The addition of LLZTO in Figure 4c further modulates the chemical environment of polymers and lithium salts, reducing the oxidation peak between 3 and 4 V. By adding SN and LLZTO, the electrochemical windows of the CSEs can be enhanced to 4.52 and 4.70 V in the LSV curves, respectively (shown in Figure 4d), which is extremely suitable for using with most commercial cathode materials.

**Figure 5:**
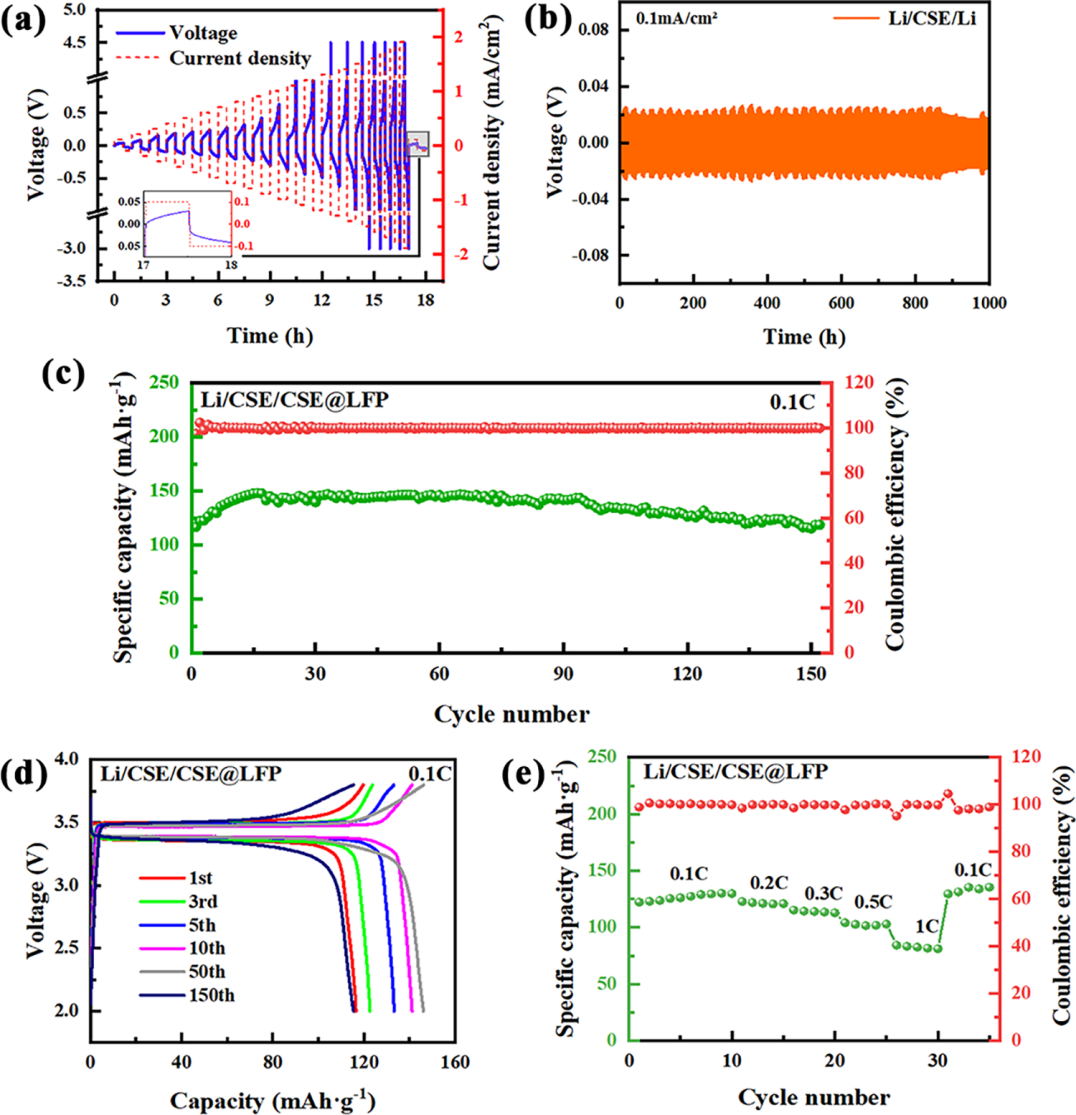
(a) Galvanostatic cycling of the Li/CSE/Li symmetric cell at step-increased current densities. (b) Long-term cycling performance of the symmetric cell at 0.1 mA/cm^2^. (c) Long-term cycling performance of the Li/CSE/CSE@LFP cell at 0.1 C. (d) Charge/discharge curves of the Li/CSE/CSE@LFP cell at 0.1 C. (e) Rate performance of the Li/CSE/CSE@LFP cell.

The inhibitory effect of the CSE membrane on the growth of lithium dendrites is studied by galvanostatic cycling of the Li/CSE/Li symmetric cell at step-increased current densities. Before the addition of LLZTO particles, the critical current density of the electrolyte reaches 1.0 mA/cm^2^ (Figure S5). As shown in Figure 5a, the critical current density of the CSE with LLZTO significantly increases to 1.5 mA/cm^2^. Moreover, after cycling at current densities ranging from 0.1 to 1.9 mA/cm^2^, the symmetric cell still maintains good stability at 0.1 mA/cm^2^, indicating that the composite electrolyte has excellent recovery performance. Figure S6 shows the EIS results after cycling at different step currents, demonstrating that the addition of LLZTO significantly improved the stability of the electrolyte. The voltage as well as the cell resistance is expected to drop suddenly if the formation of Li dendrites is uncontrollable over long cycles, indicating the short circuit of the cell. Further experiments have shown that the Li/CSE/Li symmetric cells can be stably cycled at 0.1 mA/cm^2^ for more than 1000 h without a short circuit (Figure 5b) and over 400 h even at a high current density of 0.5 mA/cm^2^ (Figure S7). The results confirm that the Li dendrite growth has been considerably restrained. The gradual increase in voltage indicates an increase in resistance, which may be related to the continuous generation of a solid electrolyte interphase (SEI) at high current densities.^59^ In conclusion, the prepared composite solid-state electrolytes have good stability toward a lithium metal anode.

Based on the excellent electrochemical performance and stability of CSEs, we assembled all-solid-state lithium-ion batteries (ASSLBs) to further evaluate their performance and practical applications. As shown in Figure 5c, the coin cell with the CSE@LFP cathode and Li metal anode was cycled over a cutoff voltage ranging from 2.0 to 3.8 V. The initial discharge specific capacity is around 116.5 mAh·g^–1^ at 0.1 C. After an activation process in the next few cycles accompanied by the formation and conversion of the interfacial layer,^62^ a high discharge specific capacity of 148.1 mAh·g^–1^ with ∼100% Coulombic efficiency is achieved. The excellent Coulombic efficiency proves that the embedding and deembedding of active Li^+^ in the electrode material is highly reversible. The capacity retention is about 90.21% after 100 cycles and 80.2% after 150 cycles. The capacity fading may be caused by the accumulation of SEI passivation layers and the possible structural and phase changes of the LFP during long-term cycles. The charge and discharge curves in Figure 5d show that the ASSLB has a smooth voltage plateau and a small overpotential of 0.1 V. Figure 5e displays the rate performance of the ASSLB; the reversible capacity decreases slightly with the increase in charge/discharge C-rate and maintains a discharge capacity over 100 mAh·g^–1^ even at 0.5 C. The excellent rate performance can be attributed to the extremely good ionic conductivity and the high migration number of lithium ions. When the current density returns to 0.1 C, the reversible capacity recovers well and remains stable, indicating that the electrolyte/electrode interface has a good electrochemical stability during rapid charge and discharge processes.

**Figure 6:**
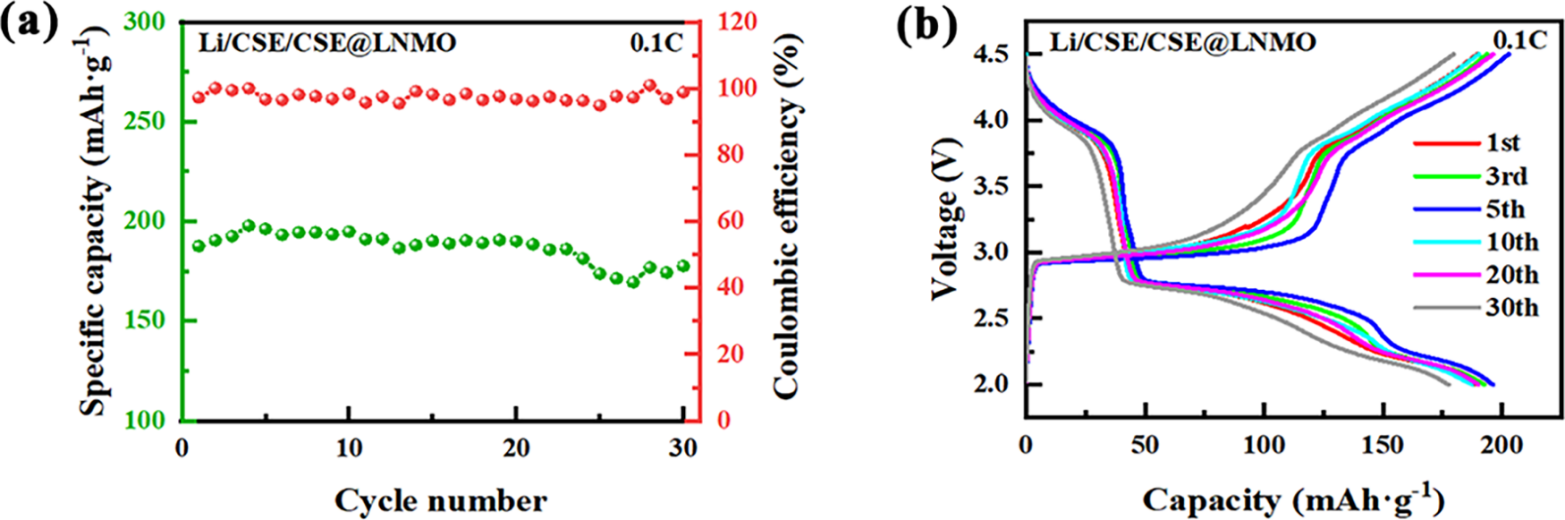
(a) Cycling performance of the Li/CSE/CSE@LNMO cell at 0.1 C. (b) Charge/discharge curves of the Li/CSE/CSE@LNMO cell over a cutoff voltage ranging from 2.0 to 4.5 V at 0.1 C.

In order to verify the operation capability of the CSE at high voltage, the ASSLBs using the high-voltage CSE@LNMO cathode and lithium metal anode were assembled. Unfortunately, as can be seen from Figure S8, irreversible redox reactions occur continuously when the Li/CSE/CSE@LNMO cell is charged to 4.8 V. Thus, the electrochemical performance of the cell is set in the voltage range from 2 to 4.5 V at 0.1 C, which is shown in Figure 6a,b. Consistent with the Li/CSE/CSE@LFP cell, the Li/CSE/CSE@LNMO cell also suffers from a short-term activation process and then can be stably cycled with the contribution of good interfacial compatibility. The discharge specific capacity is up to 197.85 mAh·g^–1^ with a capacity retention rate over 90% after 30 cycles. The decay of cell capacity is also related to the change of the LNMO structure and the increase in polarization. In addition, the high Coulombic efficiency (∼100%) throughout the cycling test reflects the excellent charge-transfer reversibility through the electrode/electrolyte interface. In conclusion, the ASSLBs work well and may have a good application prospect.

## 4 Conclusions

In this study, we prepared a composite solid electrolyte with low crystallinity, uniform phase distribution, and excellent performance by using the polymer electrolyte PVDF-HFP as the matrix, succinonitrile as the plasticizer, lithium bis(trifluoromethylsulfonyl)imide as the Li salt, and Li_6.4_La_3_Zr_1.4_Ta_0.6_O_12_ as the active filler. After a systematic exploration, we determine the optimal composition of the CSEs as 43.6% PVDF-HFP, 29.1% LiTFSI, 18.2% SN, and 9.1% LLZTO by mass fraction. The CSEs possess an ionic conductivity up to 4.05 × 10^–4^ S·cm^–1^ at room temperature and an electrochemical stability window up to 4.7 V. The Li/CSE/Li symmetric cell shows a high critical current density up to 1.5 mA/cm^2^ and can be continuously cycled for more than 1000 h at 0.1 mA/cm^2^ with a flat voltage plateau and low overpotential. The ASSLBs using the LFP cathode displays a high discharge capacity of 148.1 mAh·g^–1^ with ∼100% Coulombic efficiency and a capacity retention of 90.21% after 100 cycles. Furthermore, the CSEs were shown to be capable of serving ASSLBs in high voltage environments. Thus, it is believed that the high-performance PVDF-HFP-based CSEs with excellent interfacial compatibility is one of the most satisfactory choices for room-temperature all-solid-state lithium metal batteries.

## Supporting Information Available

Equivalent circuit of the SS/CSE/SS symmetrical cell (Figure S1); ionic conductivity of the CSE membrane at different temperatures (Figure S2); CV curves of the GPE-a membrane at the scan rate ν = 0.5 mV·s^–1^ (Figure S3); CV curves of the GPE-b membrane at the scan rate ν = 0.5 mV·s^–1^ (Figure S4); cycling curves of the Li/GPE-b/Li symmetric cell at different current densities (Figure S5); electrochemical impedance spectroscopy of GPE-b and CSE during the current step test (Figure S6); long-term cycling curve of the Li/CSE/Li cell at 0.5 mA/cm^2^ (Figure S7); and charge/discharge curves of the Li/CSE/CSE@LNMO cell over a cutoff voltage ranging from 2.0 to 4.8 V at 0.1 C (Figure S8) (PDF)
