# Supplementary material for: High-Performance Poly(vinylidene fluoride-hexafluoropropylene)-Based Composite Electrolytes with Excellent Interfacial Compatibility for Room-Temperature All-Solid-State Lithium Metal Batteries
Source: ACS Omega. 2022 May 30;7(23):19631–9. doi: 10.1021/acsomega.2c01338 (PMC9202062; doi:10.1021/acsomega.2c01338)
Supplement: Supplementary file 1 — ao2c01338_si_001.pdf [file ao2c01338_si_001.pdf]

Supporting Information

High-performance PVDF-HFP based composite electrolyte with excellent interfacial compatibility for room-temperature all-solid-state lithium metal battery

Si-Yuan Du,[1,3‡] *Guo-Xi Ren,*[1‡] *Nian Zhang,*[1*] *and Xiao-Song Liu*[1,2,4,5*]

[1]State Key Laboratory of Functional Materials for Informatics, Shanghai Institute of Microsystem and Information Technology, Chinese Academy of Sciences, Shanghai 200050, China

[2] Tianmu Lake Institute of Advanced Energy Storage Technologies, Liyang, Jiangsu 213300, China

[3] University of the Chinese Academy of Sciences, Beijing 100049, China

[4] School of Physical Science and Technology, Shanghai Tech University, Shanghai 201210, China

[5] National Synchrotron Radiation Laboratory, University of Science and Technology of China, Hefei, Anhui 230029, China

*E-mail: zhangn@mail.sim.ac.cn (N.Z.)

*E-mail: xliu3@mail.sim.ac.cn (X.-S.L.)

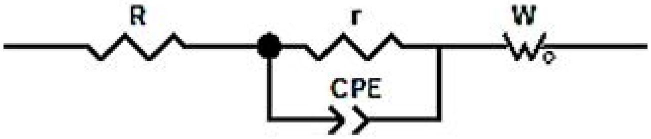

Figure S1. The equivalent circuit of the SS/CSE/SS symmetrical cell.

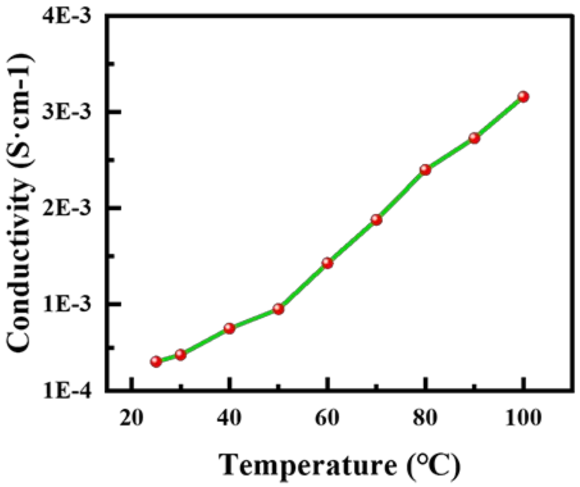

Figure S2. The ionic conductivity of the CSE membrane at different temperatures.

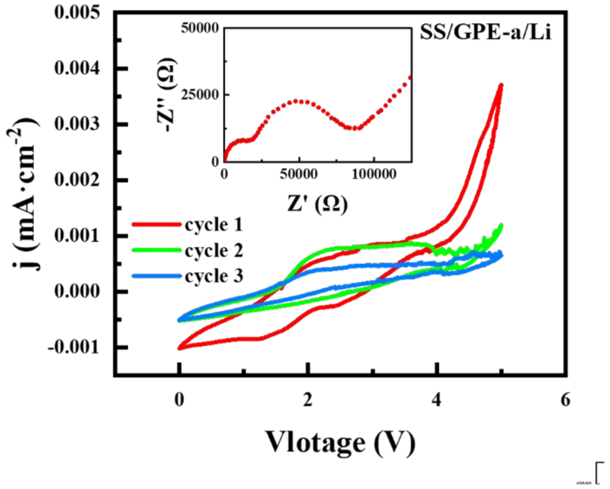

Figure S3. The CV curves of GPE-a membrane at the scan rate $\nu = 0.5\ mV{\cdot}s^{-1}$.

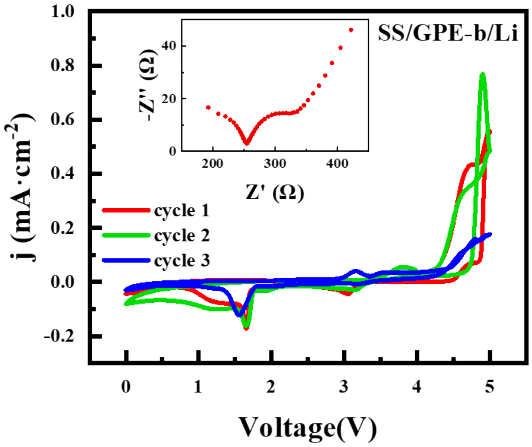

Figure S4. The CV curves of GPE-b membrane at the scan rate ν = 0.5 mV·s^{-1}.

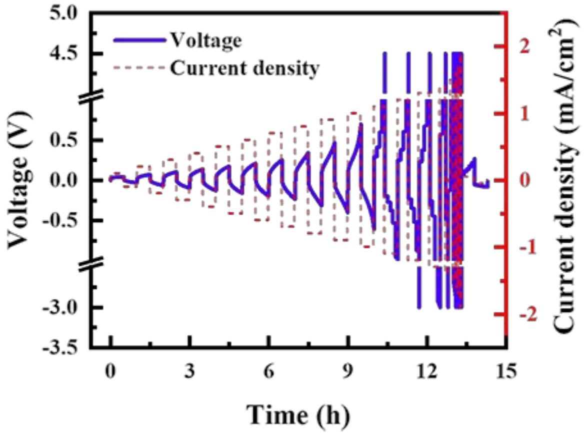

Figure S5. Cycling curves of the of the Li/GPE-b/Li symmetric cell at different current densities.

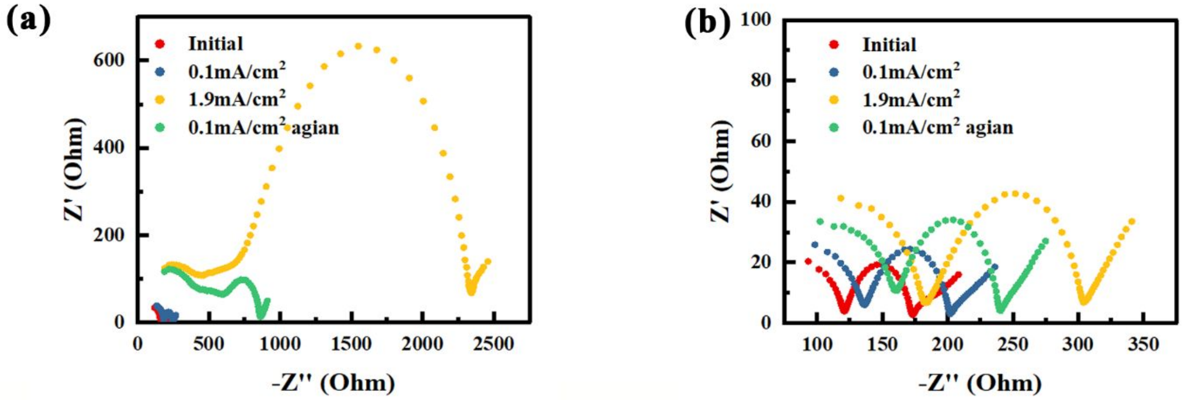

Figure S6. Electrochemical impedance spectroscopy of (a) GPE-b and (b) CSE during the current step test.

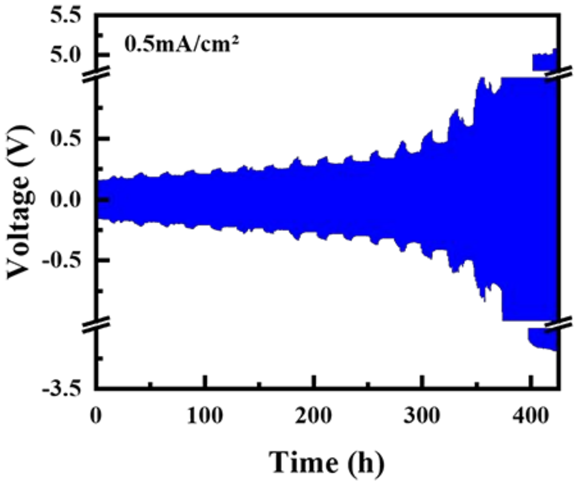

Figure S7. Long-term cycling curve of the Li/CSE/Li cell at 0.5 mA/cm².

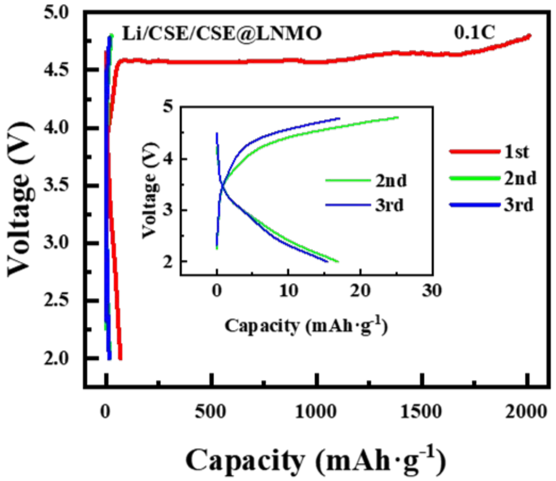

Figure S8. Charge/discharge curves of the Li/CSE/CSE@LNMO cell over a cut-off voltage ranging from 2.0 V to 4.8 V at 0.1 C.
